# Missense mutations in small muscle protein X-linked (*SMPX*) cause distal myopathy with protein inclusions

**DOI:** 10.1007/s00401-021-02319-x

**Published:** 2021-05-11

**Authors:** Mridul Johari, Jaakko Sarparanta, Anna Vihola, Per Harald Jonson, Marco Savarese, Manu Jokela, Annalaura Torella, Giulio Piluso, Edith Said, Norbert Vella, Marija Cauchi, Armelle Magot, Francesca Magri, Eleonora Mauri, Cornelia Kornblum, Jens Reimann, Tanya Stojkovic, Norma B. Romero, Helena Luque, Sanna Huovinen, Päivi Lahermo, Kati Donner, Giacomo Pietro Comi, Vincenzo Nigro, Peter Hackman, Bjarne Udd

**Affiliations:** 1 Folkhälsan Research Center, Helsinki, Finland; 2 Department of Medical Genetics, Medicum, University of Helsinki, Helsinki, Finland; 3 Neuromuscular Research Center, Fimlab Laboratories, Tampere University and University Hospital, Tampere, Finland; 4 Neuromuscular Research Center, Department of Neurology, Tampere University and University Hospital, Tampere, Finland; 5 Division of Clinical Neurosciences, Department of Neurology, Turku University Hospital, Turku, Finland; 6 Dipartimento di Medicina di Precisione, Università degli Studi della Campania “Luigi Vanvitelli”, Naples, Italy; 7 Section of Medical Genetics, Mater Dei Hospital, Msida, Malta; 8 Department of Anatomy and Cell Biology, Faculty of Medicine and Surgery, University of Malta, Msida, Malta; 9 Neuroscience Department, Mater Dei Hospital, Msida, Malta; 10 Neuromuscular Disease Center AOC, University Hospital Nantes, Nantes, France; 11 IRCCS Foundation Ca’ Granda Ospedale Maggiore Policlinico, Neurology Unit, Milan, Italy; 12 Department of Neurology, University Hospital Bonn, Bonn, Germany; 13 AP-HP, Institute of Myology, Centre de Référence des Maladies Neuromusculaires, Hôpital Pitié-Salpêtrière, Paris, France; 14 Neuromuscular Morphology Unit, Institute of Myology, Myology Research Centre INSERM, Sorbonne Université, Hôpital Pitié-Salpêtrière, Paris, France; 15 Department of Pathology, Fimlab Laboratories, Tampere University Hospital, Tampere, Finland; 16 Institute for Molecular Medicine Finland FIMM, Technology Centre, University of Helsinki, Helsinki, Finland; 17 IRCCS Fondazione Ca’ Granda Ospedale Maggiore Policlinico, Neuromuscular and Rare Disease Unit, Milan, Italy; 18 Dino Ferrari Center, Department of Pathophysiology and Transplantation, University of Milan, Milan, Italy; 19 Telethon Institute of Genetics and Medicine (TIGEM), Pozzuoli, Italy; 20 Department of Neurology, Vaasa Central Hospital, Vaasa, Finland

**Keywords:** X-linked, Distal myopathy, Proteinopathy, Amyloidogenesis, Stress granules

## Abstract

Using deep phenotyping and high-throughput sequencing, we have identified a novel type of distal myopathy caused by mutations in the Small muscle protein X-linked (*SMPX*) gene. Four different missense mutations were identified in ten patients from nine families in five different countries, suggesting that this disease could be prevalent in other populations as well. Haplotype analysis of patients with similar ancestry revealed two different founder mutations in Southern Europe and France, indicating that the prevalence in these populations may be higher. In our study all patients presented with highly similar clinical features: adult-onset, usually distal more than proximal limb muscle weakness, slowly progressing over decades with preserved walking. Lower limb muscle imaging showed a characteristic pattern of muscle involvement and fatty degeneration. Histopathological and electron microscopic analysis of patient muscle biopsies revealed myopathic findings with rimmed vacuoles and the presence of sarcoplasmic inclusions, some with amyloid-like characteristics. In silico predictions and subsequent cell culture studies showed that the missense mutations increase aggregation propensity of the SMPX protein. In cell culture studies, overexpressed SMPX localized to stress granules and slowed down their clearance.

**Supplementary Information:**

The online version contains supplementary material available at 10.1007/s00401-021-02319-x.

## Introduction

Distal myopathies are a clinically, histopathologically and genetically heterogeneous group of inherited skeletal muscle diseases. In some entities the muscle weakness remains restricted to the distal muscles, and in others involvement of the proximal muscles may occur as the disease progresses [26]. Over the years, deep phenotyping, including muscle imaging and histopathological evidence, and molecular genetics have resulted in the identification of more than 25 genes related to distal myopathies [33]. Advancements in high-throughput sequencing (HTS) have increased the rate of molecular diagnosis for families with inherited rare neuromuscular disorders. However, over 40% of patients, in particular singletons, still remain without conclusive molecular diagnosis often due to lack of sufficient family data and material [12, 32]. To consider pathogenicity of previously unknown genes as causative for a neuromuscular disease, either more than one family with similar phenotype or a very large family is needed.

Using deep phenotyping, HTS and subsequent functional studies, we describe here a novel adult-onset distal myopathy observed in ten patients sharing common clinical features, characteristic muscle imaging (MRI) features, histopathological findings and missense variants in the novel myopathy gene *SMPX*.

SMPX (also known as Chisel, CSL) is a proline-rich protein of 88 amino acids (9 kDa). It is predominantly expressed in skeletal muscles and heart, with a costameric and intermyofibrillar localization and highest expression in slow muscle fibers [29]. When overexpressed in mouse myoblasts, it associates with focal adhesion proteins, promotes myoblast fusion, and modulates actin turnover and cell shape upstream of Rac1 and p38 [29, 35]. While mutations causing total loss of SMPX have been reported to cause hearing loss, its molecular role and function in skeletal muscle remains largely unknown.

Our functional studies show that the four different pathogenic missense mutations in *SMPX* observed in our patients make the mutant SMPX protein aggregation prone. The pathological effect is observed as prominent sarcoplasmic protein inclusions in the muscle fibers of patients.

## Materials and methods

### Patients and clinical examinations

**Fig. 1:**
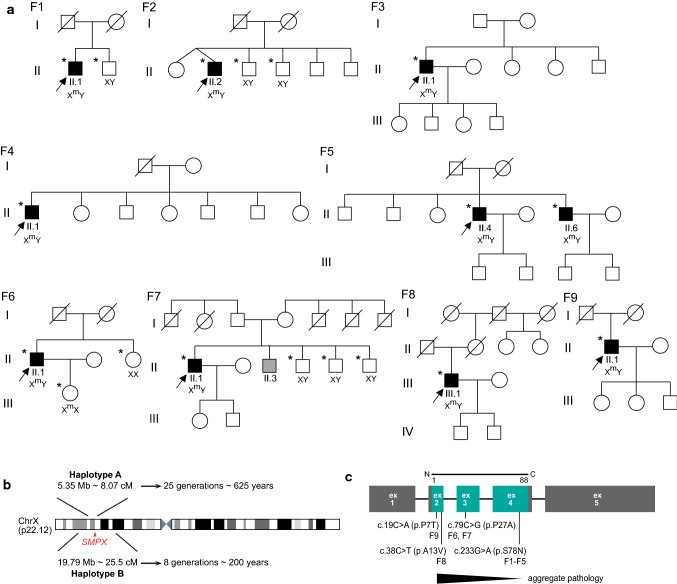
**a** Pedigrees of the families included in the study. DNA samples were collected from the individuals marked with an *asterisk*. Corresponding gentoypes are displayed for these individuals where X^m^ represents a mutated allele and X represents a wild-type allele. F7 II.3 is indicated as suffering from poliomyelitis and thus was not included in the study. **b** Haplotype analysis, showing the Haplotype A (Italian/Maltese haplotype) observed in Families F1–F2 and Haplotype B (French haplotype) observed in F6–F7 along with the corresponding age of the haplotypes. **c** A summary of the identified missense mutations in *SMPX* and their correlation with the observed phenotypes in F1–F9

All patients underwent clinical neurological examination. Besides the 10 affected patients, we collected blood samples for DNA analysis from 8 asymptomatic family members from 9 families (Fig. 1a). All muscle biopsies were obtained for diagnostic purposes after informed consent, and studies were approved by institutional review boards. The study was performed according to the Declaration of Helsinki.

Ethical approval for this study falls under HUS:195/13/03/00/11. Informed consent from the patients was obtained at the time of sample collection.

**Fig. 2:**
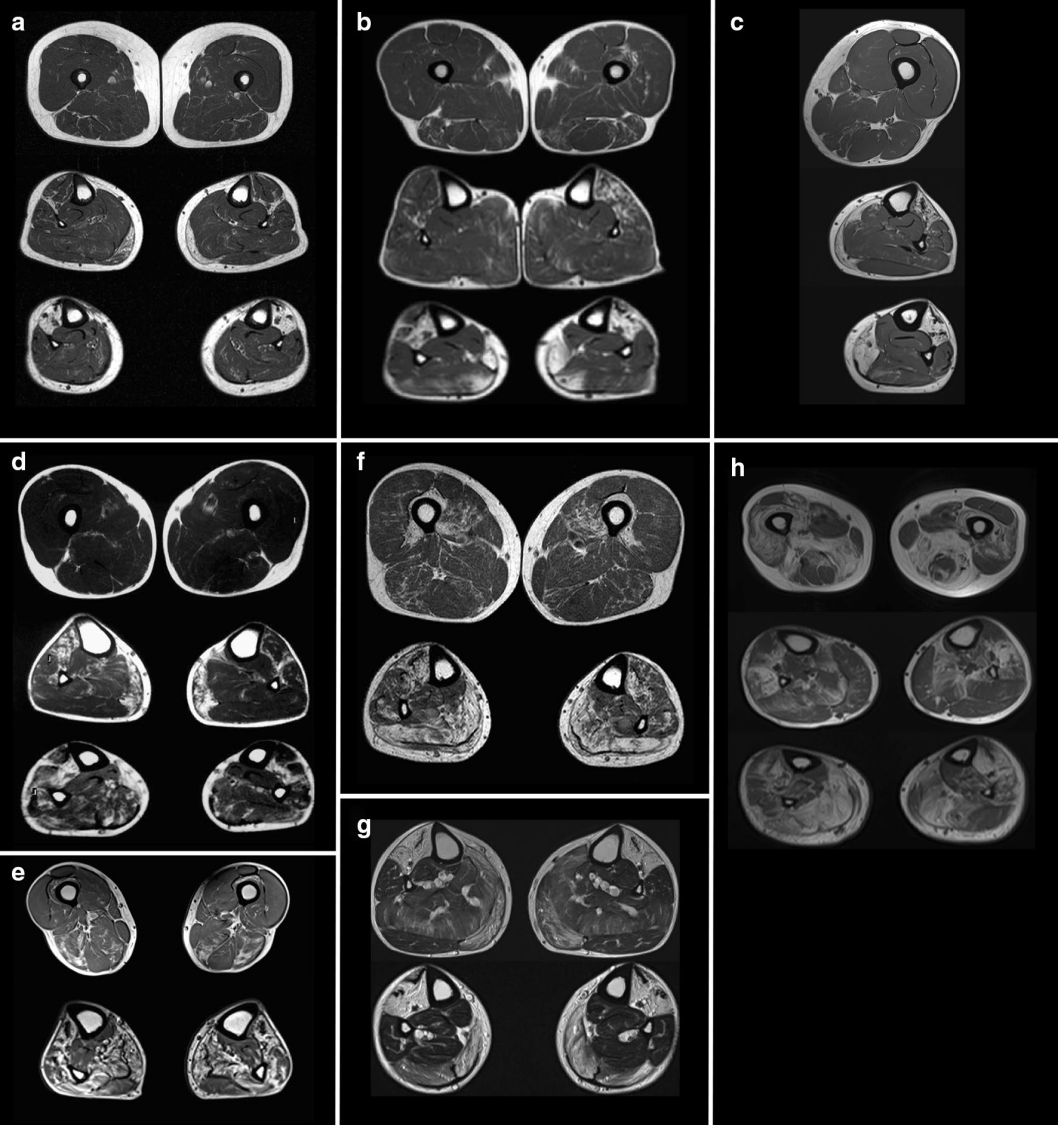
Magnetic resonance imaging (MRI) T1 sequences of patients with SMPX distal myopathy. **a** Patient F1 II.1 at 57 yrs showing normal thighs but fatty degeneration in lower legs: anterior compartment and medial gastrocnemius muscles. **b** F2 II.2 at 61 yrs showing minor degenerative change in thigh muscles semimembranosus, biceps femoris and left vastus intermedius; fatty degenerative changes in the anterior compartment muscles more on the left of proximal lower leg and fatty replacement of anterior compartment and part of the soleus muscles in the distal lower legs. **c** F3 II.1 at 58 yrs (left lower limb) with normal thigh and severe fatty degeneration in lower leg anterior compartment, medial gastrocnemius and medial part of distal soleus. **d** F6 II.1 at 52 yrs showing normal thigh and fatty degenerative changes in the anterior compartment (more on the right) and medial gastrocnemius muscles of proximal lower leg, and anterior compartment with soleus muscles in the distal lower legs. **e** F6 II.1 at 60 yrs with early degenerative changes in biceps femoris and semimembranosus in the thigh and more fatty degeneration of anterolateral compartments and of medial gastrocnemius and soleus in the lower legs. **f** F7 II.1 at 56 yrs shows milder fatty degeneration in vastus intermedius and medialis, semimembranosus and biceps femoris on the thigh, and in both posterior and anterior compartments on the lower legs, less in the lateral peroneal muscles. **g** F8 III.1 at 58 yrs shows lower legs with severe fatty replacement of anterior compartments and medial heads of gastrocnemius and milder changes in the soleus. **h** F9 II.1 at 76 yrs showing pronounced fatty degeneration in thighs: more in hamstrings than the lateral and intermediate vastus of the quadriceps, severe changes in distal lower leg anterior compartment and soleus muscles and peroneals more on the right

Muscle imaging data, electrophysiological examination results (nerve conduction studies and needle electromyogram, EMG), creatine kinase (CK) measurements and cardiac function test results were obtained in most patients (Table 1). Muscle MRI findings (Fig. 2) with axial sections of the lower limb muscles were evaluated in 8 patients, in two of whom (F2 II.2 and F6 II.1) also the shoulder girdle/upper limb muscles were imaged. Echocardiography was performed in 9 patients.

**Table 1:** Clinical, histopathological and MRI details of patients included in the study. Identified mutations are written in human genome variation society (HGVS) nomenclature

Patient ID	F1 II.1	F2 II.2	F3 II.1	F4 II.1	F5 II.4	F5 II.6	F6 II.1	F7 II.1	F8 III.1	F9 II.1
SMPX mutation NM_014332.3 (NP_055147.1)	c.233G > A (p.S78N)	c.233G > A (p.S78N)	c.233G > A (p.S78N)	c.233G > A (p.S78N)	c.233G > A (p.S78N)	c.233G > A (p.S78N)	c.79C > G (p.P27A)	c.79C > G (p.P27A)	c.38C > T (p.A13V)	c.19C > A (p.P7T)
Age of onset (years)/first symptoms	26/finger extensor weakness > ankle, toe dorsiflexion	20/mild lower limb weakness	30/distal leg weakness	54/distal leg weakness	40/distal leg weakness	50/distal leg weakness	48/pain in calves	43/distal lower leg weakness	43/asymmetric ankle dorsiflexion weakness	60/progressive distal limb weakness
Age at examination/disease duration (years)	52/26	61/41	57/27	58/4	55/15	57/7	62/14	61/18	67/24	77/17
Distal upper limb weakness (normal, mild, moderate or severe) (mild = MRC 4, moderate = 2–3, severe = 0–1)	Severe/extensor prominent	Mild/extensor prominent	Mild/extensor prominent	Moderate/extensors and intrinsics	Moderate/extensor prominent	No weakness	Moderate/extensor and intrinsics	No weakness	No weakness	Severe/extensor prominent
Proximal upper limb weakness (no, mild, moderate or severe)	No weakness	Moderate	Mild	Mild	Severe	Mild	No weakness	No weakness	No weakness	Moderate
Proximal lower limb weakness (no, mild, moderate or severe)	No weakness	Mild	No weakness	No weakness	No weakness	No weakness	No weakness	Mild	No weakness	Moderate
Distal lower limb weakness (no, mild, moderate or severe)	Severe, anterior prominent	Moderate, anterior prominent	Moderate, anterior prominent	Severe, anterior prominent	Severe, anterior prominent	Severe, anterior prominent	Severe, anterior prominent	Severe, anterior prominent	Severe, anterior prominent	Severe, anterior prominent
Scapular winging	None	None	Yes	Yes	Yes	Yes	None	None	None	Prominent
Respiratory involvement	No	No	No	No	No	No	No	No	No	No
Spirometry/FVC value (if performed)	Normal	Normal	NA	NA	NA	NA	Normal; FVC 112%	Normal	Normal	NA
Cardiomyopathy by ultrasound	No	No	No	No	No	No	No	No	No	NA
Cataracts	No	No	No	No	No	No	No	No	No	No
Ambulant at least for short distances when last examined	Yes	Yes	Yes	Yes	Yes	Yes	Yes	Yes	Yes	Yes
Documented hearing impairment	No	No	No	No	No	No	No	No	No	No
CK	1.5 UNL	1–1.5 UNL	1.5 UNL	~ 2.5 UNL	~ 2.5 UNL	2 UNL	1–1.5 UNL	2 UNL	Normal	~ 2.5 UNL
EMG	Myopathic	Myopathic	Myopathic	Myopathic	Myopathic	Myopathic	Myopathic	Myopathic	Mixed neurogenic-myopathic	Myopathic
Histological findings	RV, few SI, MF, size variability, few fetal myosin reactive necrotic fibers	Some central nuclei, few RV, mild size variability	NA	Fiber size variability, internal nuclei, some cox neg fibers	NA	NA	RV, SI filamentous smaller peripheral and central larger with autophagic material	Fibrosis, internal nuclei, RV, few necrotic fibers with mononuclear infiltrate	SI, MF, fiber size variability, eosinophilic protein aggregates	RV, SI, MF, fiber size variability
Muscle imaging (MRI)	Normal thighs Lower leg: fatty degeneration in anterior compartment and medial gastrocnemius	Fatty degeneration: semimembranosus, biceps femoris and left vastus intermedius Lower legs: anterior compartment and part of the soleus muscles in the: periscapular and deltoid muscles, severe involvement of paraspinal muscles	Normal thigh Lower leg: severe fatty degeneration in lower leg anterior compartment, medial gastrocnemius and medial part of distal soleus	NA	Fatty degeneration: mild-moderate in proximal and severe in distal lower limb muscles	NA	52 yrs: normal thighs Lower legs: fatty degeneration in the anterior compartment (more on the right), medial gastrocnemius and soleus 60 yrs: also biceps femoris and semimembranosus involvement	Thighs: mild fatty degeneration in vastus medialis and intermedius,semimembranosus and biceps femoris Lower legs: posterior and anterior compartment, less in the peroneal muscles	Thighs: normal Lower legs: severe fatty replacement of anterior compartments and milder changes in medial heads of gastrocnemius (right > left) and soleus	Hamstrings and quadriceps, severe changes in distal lower leg anterior compartment and soleus muscles

*NA* not assessed; *RV* rimmed vacuoles; *SI* sarcoplasmic inclusions; *MF* myofibrillar disarray

### Muscle biopsy and immunohistochemical studies

Snap-frozen muscle samples were obtained from seven probands and processed with routine muscle histopathological procedures, including hematoxylin & eosin, modified Gomori’s trichrome, NADH tetrazolium reductase, and COX-SDH stainings [4]. Immunofluorescent analysis using standard methodology was performed using the following primary antibodies: rabbit polyclonal anti-SMPX (PA3-070, Thermo Fisher Scientific, RRID:AB_2540497), rabbit polyclonal anti-LC3b (2775, Cell Signaling Technology, RRID:AB_915950) rabbit polyclonal anti-p62 (P0067, Sigma-Aldrich, RRID:AB_1841064), mouse monoclonal anti-TDP-43 (clone 2E2-D3, Sigma-Aldrich, RRID:AB_10806030), mouse monoclonal anti-αB-crystallin (ABCrys-512, Leica Biosystems, RRID:AB_442024), mouse monoclonal anti-vinculin (ab130007, Abcam, RRID:AB_11156698), rabbit polyclonal anti-BAG3 (10599-1-AP, Proteintech, RRID:AB_2062602), rabbit polyclonal anti-HSPB8 (ab79784, Abcam, RRID:AB_1603573), rabbit monoclonal anti-TIAL1 D32D3 (8509, Cell Signaling Technology, RRID:AB_10839263) and goat polyclonal anti-eIF3η (N-20, sc-16377, Santa Cruz Biotechnology, RRID:AB_671941). Alexa Fluor 488/546 -conjugated secondary antibodies (Thermo Fisher Scientific) were used for detection, and nuclei were counterstained with Hoechst. In addition, DAB immunostaining was performed using mouse monoclonal anti-myotilin (NCL-MYOTILIN, Leica Biosystems, RRID:AB_563903), mouse monoclonal anti-desmin (MU 072-UC, BioGenex), mouse monoclonal anti-phosphorylated neurofilament H (clone SMI-31, BioLegend, RRID:AB_2564641), and rabbit polyclonal anti-ubiquitin (Z458, Agilent/Dako, RRID:AB_2315524). Menadione and alkaline Congo red [9] stainings were performed on patient F9 II.1. Staining with Amytracker 680 (Ebba Biotech AB, Solna, Sweden) was performed on patient F8 III.1. Microscopic images were obtained using the Zeiss Axio Imager M2 system (Carl Zeiss AG) or a Leica TCS SP8 confocal microscope (Leica Microsystems). Ultrathin sections were prepared for electron microscopy and examined with a JEOL 1400 transmission electron microscope (JEOL, Japan). Electron micrographs were obtained using the Olympus-SIS Morada digital camera (Olympus Soft Imaging Solutions, Münster, Germany).

### Molecular genetic analyses

Genomic DNA from probands and available and informative family members (Fig. 1a) was isolated from blood cells using standard techniques.

The proband F1 II.1 underwent Exome Sequencing (ES) using SeqCap EZ Human Exome Library v2.0 (Roche, USA) at FIMM (Helsinki, Finland). Raw reads were aligned using BWA on UCSC hg19 reference genome and variants were called according to GATK recommendations. Variant annotation was done using Annovar. ES results were first filtered on standard quality parameters and then using a minor allele frequency of 0.001 in ExAC and gnomAD databases. Thereafter, we focused on unique variants in a list of muscle-expressed genes from the skeletal muscle transcriptome [21, 38, 40]. Upon identification of a novel variant, *SMPX* was included as a candidate gene in the revised MYOcap [5] and Motorplex [31] gene panels. Probands of F2–F9 underwent sequencing using MYOcap/Motorplex targeted gene panels.

*SMPX* variants are annotated following the reference sequences: NM_014332.3 (transcript) and NP_055147.1 (protein). PrimateAI [37], CADD, MutationTaster, SIFT and PolyPhen2 were used to assess the in silico pathogenicity of the variants, and SpliceAI [14] was used to predict their potential effects on splicing. Variant pathogenicity was evaluated according to the ACMG-AMP guidelines and revised suggestions made in case of lack of available family members for cosegregation [15]. Validation and Sanger sequencing of *SMPX* variants was done by PCR. SNP genotyping was performed on Illumina Infinium Global Screening Array + MD v 2 (GSAMD-24v2), with 759,993 markers using DNA samples from family members in F1, F2 and F3, F4 for haplotype analysis.

### Plasmid constructs

Wild-type human *SMPX* ORF was cloned to pCDNA3.1/V5-His-TOPO (Thermo Fisher), to produce a C-terminally V5/His6-tagged SMPX construct (SMPX-V5). Missense mutations were introduced to the construct by site-directed mutagenesis. Wild-type and mutant SMPX were then cloned in frame with an N-terminal Myc tag to pSBtet-Hyg [18] (a gift from Eric Kowarz; Addgene plasmid # 60,508; RRID:Addgene_60508) to produce pSBtet-Hyg-Myc-SMPX constructs.

### Aggregation prediction

The effect of the variants on amyloid aggregation of SMPX was predicted using the PASTA 2.0 server (http://old.protein.bio.unipd.it/pasta2/) [39].

### Analysis of SMPX solubility

To evaluate the solubility of the SMPX protein, wild-type and mutant SMPX-V5 constructs were expressed in HeLa cells for 3 d. The cells were pelleted in PBS, lysed in RIPA buffer (50 mM Tris–HCl pH 8.0, 150 mM NaCl, 1% Triton X-100, 0.5% sodium deoxycholate, 0.1% SDS) supplemented with 1 mM MgCl_2_, 1× HALT Protease Inhibitor Cocktail (Thermo Fisher) and 25 U/ml of Pierce Universal Nuclease for Cell Lysis (Thermo Fisher) by rotating 60 min at 8 °C, and triturated through a 27G needle. Supernatant and pellet fractions were separated by centrifugation (16,000*g*, 15 min at 4 °C). The pellets were washed once with RIPA buffer and centrifuged as above. The samples were combined with 2× SDS sample buffer with 10% 2-mercaptoethanol and heated 5 min at 95 °C and analyzed by western blotting using antibodies against the V5 tag (Thermo Fisher R960-25, RRID:AB_2556564), tubulin (YL1/2, Abcam ab6160, RRID:AB_305328), and histone 3 (Abcam ab1791, RRID:AB_302613). Total protein was stained with the Revert 700 Total Protein Stain (LI-COR, Lincoln, NE, USA) and detected with an Odyssey scanner.

### Microscopic analyses of HeLa cells

For microscopic analyses, HeLa cells were plated on coverslips in 24-well plates and transfected with SMPX-V5 constructs (500 ng/well) using FuGENE 6. Three days after transfection, the cells were treated with 20 µM MG132 (Sigma-Aldrich) for 2 h or left untreated. Cells were washed with PBS and fixed with 4% PFA for 15 min. Immunofluorescence stainings were performed with the following antibodies: V5 mouse mAb (RRID:AB_2556564), V5 rabbit pAb (Sigma-Aldrich AB3792, RRID:AB_91591), TIA1 goat pAb (Abcam ab61700, RRID:AB_945832), TIAL1 rabbit pAb (Abcam ab26257, RRID:AB_470826), TIAL1 rabbit mAb D32D3 (RRID:AB_10839263), G3BP mouse mAb (Abcam ab56574, RRID:AB_941699), HNRNPA1 mouse mAb 9H10 (Abcam ab5832, RRID:AB_305145), eIF3η goat pAb (RRID:AB_671941), and Oligomer A11 rabbit pAb (Thermo Fisher AHB0052, RRID:AB_2536236). Nascent proteins were labeled and detected with the Click-iT Plus OPP Alexa Fluor 594 Protein Synthesis Assay Kit (Thermo Fisher) according to the manufacturer’s instructions. Images were acquired with Zeiss Axio Imager M2 using 40× NA 1.30 and 20× NA 0.80 objectives, or with a Leica TCS SP8 confocal microscope using a 40× NA 1.10 objective (Leica Microsystems).

### Quantitative SG analyses

For SG quantitation experiments, HeLa cells were cultured on coverslips or 96-well plates (ViewPlate-96, PerkinElmer) and transfected with SMPX-V5 constructs. Three days after transfection, the cells were left non-treated, fixed immediately after stress treatment (20 µM MG132 2 h or 500 µM sodium arsenite 45 min), or washed once with warm culture medium and recovered in fresh medium for 1 or 3 h after stress. Cells were fluorescently stained with V5 and TIAL1 antibodies and Hoechst, and imaged with a 20× NA 0.80 objective on Zeiss Axio Imager M2 (MG132 experiments; coverslips) or a 20× NA 0.40 objective on the ImageXpress Pico instrument (arsenite experiments; 96-well plates). Automated image analysis was performed using the CellProfiler 4.0.5 software [25]. Briefly, SMPX-transfected and untransfected cells were identified based on V5 staining intensity, and cytoplasmic TIAL1 spots were identified as SGs. In V5-positive cells, median V5 signal intensity of the SGs and the surrounding donut of 5 pixels was determined, and those with the intensity ratio > 1.2 were classified as V5-enriched. Data were collected from four independent experiments, and group means (proportion of SG-containing cells and mean number of SGs per analyzed cell) from the replicate experiments were compared using two-way repeated-measures ANOVA or repeated-measures mixed model test with the Geisser–Greenhouse correction and Dunnett’s multiple correction test.

### C2C12 cell experiments

C2C12 myoblasts were cultured in growth medium (pyruvate-free DMEM with 20% FCS, Glutamax, and penicillin/streptomycin). For differentiation into myotubes, myoblasts were grown to confluency on collagen-coated dishes and then cultured 3 d in differentiation medium (pyruvate-free DMEM with 2% heat-inactivated horse serum, L-glutamine, and penicillin/streptomycin) supplemented with 10% OPTI-MEM I (Thermo Fisher Scientific), and additional 1–2 d in differentiation medium alone.

In transient transfection experiments, subconfluent C2C12 myoblasts were transfected with SMPX-V5 constructs using Lipofectamine 3000 (Thermo Fisher Scientific), and differentiation was started on the following day.

To create C2C12 cells with inducible expression of Myc-SMPX, C2C12 myoblasts were cotransfected with wild-type or mutant pSBtet-Hyg-Myc-SMPX and pCMV(CAT)T7- SB100 [24] (a gift from Zsuzsanna Izsvak; Addgene plasmid # 34879, RRID:Addgene_34879) in a 9:1 ratio using Lipofectamine 3000. Polyclonal pools of stably transfected cells were selected in growth medium with 200 µg/ml hygromycin B (Thermo Fisher Scientific) and maintained in growth medium with 50 µg/ml hygromycin B. Myc-SMPX expression in myoblasts or differentiating myotubes was induced with 10 µg/ml doxycycline 2 days before fixing. Heat shock (45 °C 1 h) was used to induce SG formation in myoblasts.

IF stainings were performed with V5 mouse mAb (RRID:AB_2556564), TIAL1 rabbit mAb D32D3 (RRID:AB_10839263), and Myc-tag (9B11) mouse mAb (2276, Cell Signaling Technology, RRID: AB_331783).

## Results

### Clinical findings

In all ten families, except the two brothers in F5, all probands reported negative family history of muscle weakness and presented as sporadic cases (Fig. 1a). In the probands of families F1–F5, onset of finger extensor/ankle dorsiflexor weakness was observed in the 3rd–4th decade. Thereafter, the symptoms slowly progressed over decades to severe distal more than proximal upper and lower limb weakness (Table 1). The observed clinical phenotype was consistent with some individual variations as seen in the probands of F6–F9, and remarkably with retained walking capacity even in the oldest patient in F9 at the age of 80 years. Cardiac evaluations with echocardiography were normal and no hearing loss was recorded even at higher age (Table 1). Muscle imaging of the patients showed a characteristic MRI pattern of muscle involvement (Fig. 2) with fatty replacement in the anterior compartment muscles of the lower legs, later in the calf muscles medial gastrocnemius and soleus, and very late changes in the thigh muscles more in the hamstrings.

### Molecular genetics

We analyzed the HTS data from the probands for potential disease-causing variants that could explain their disease phenotype but did not identify causative variants in any of the known neuromuscular disease genes. Prioritizing unique variants in genes expressed in skeletal muscles [21, 38, 40] but not known to cause muscle disease, we observed the same variant c.233G > A (p.S78N) in the *SMPX* gene first in F1 and later in all probands of F2–F5. The variant was not present in any of the available healthy family members (Fig. 1a). Three additional *SMPX* variants (Fig. 1c) were identified after including *SMPX* as a candidate in our gene panels [5, 31] in the probands of four additional families: c.79C>G (p.P27A) in families F6 and F7 with French ancestry, c.38C>T (p.A13V) in the German family F8, and c.19C>A (p.P7T) in the Finnish family F9. None of the identified mutations were predicted to affect splicing by SpliceAI [14].

The observed mutations were absent in public genome aggregation databases except for the very late onset c.19C>A (p.P7T), which has a population frequency of 2.92 × 10^–5^ (6/205,256 alleles) in the Genome Aggregation Database (gnomAD). Families F1–F5 shared ancestral history tracing back to Malta. SNP genotyping of F1 and F2 revealed a common haplotype of chrX:17499443–22849591 amounting to 5.35 Mb (Fig. 1b). The shared haplotype spanned a mean length of 8.07 cM and was estimated to be approximately 25 generations or 625 years old [8, 30]. The French families F6–F7 shared in chrX:12750254–32544555 a haplotype of 19.79 Mb (25.5 cM), going back to approximately eight generations or 200 years (Fig. 1b).

### Histopathology, immunohistochemistry and electron microscopy (EM)

**Fig. 3:**
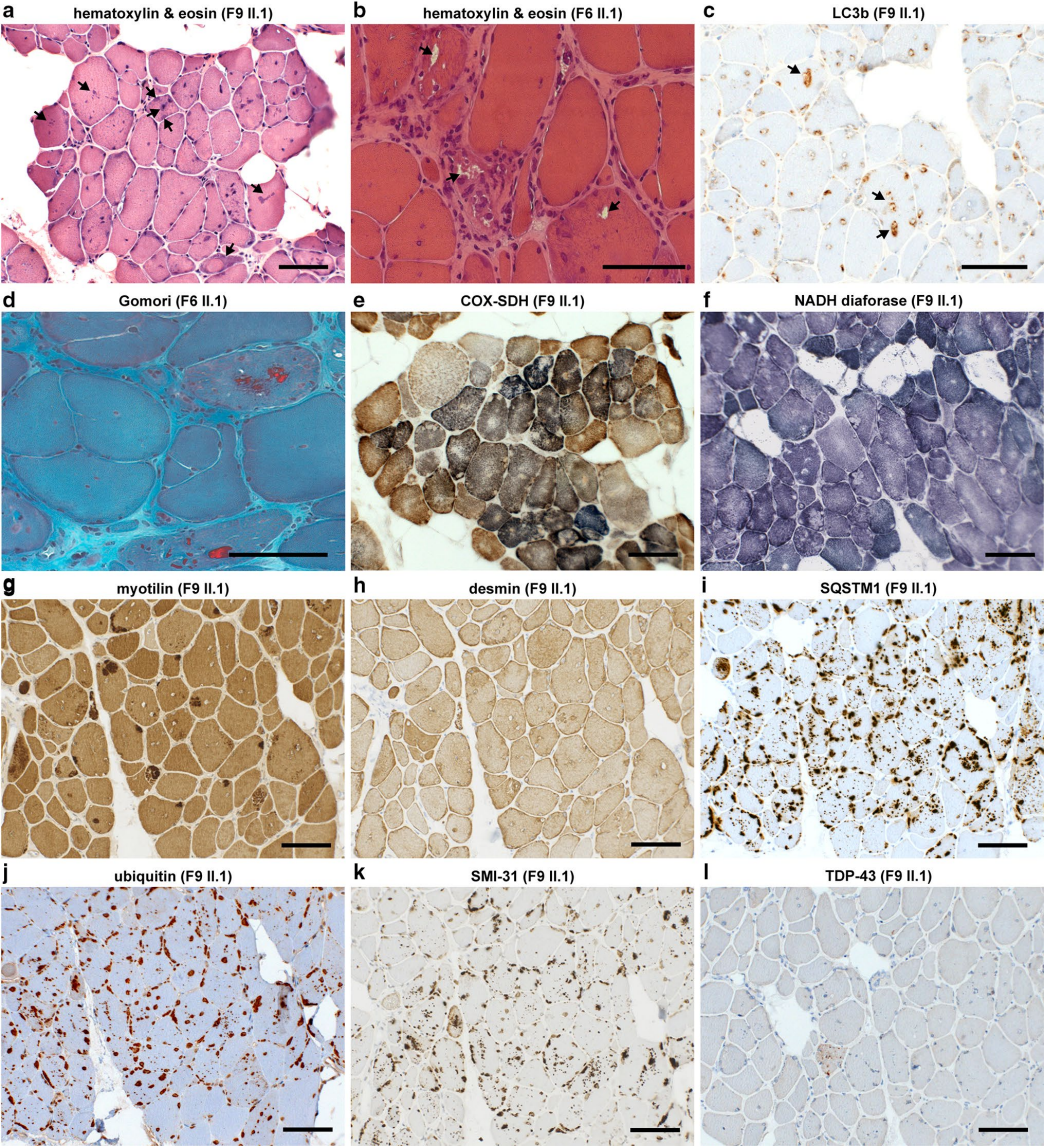
Histochemical and DAB immunohistochemical stainings. **a** Hematoxylin & eosin (HE) staining shows fiber size variation and internal nuclei, multiple cytoplasmic inclusions are present showing sarcoplasmic and subsarcolemmal localization (*arrows*). **b**, **c** Rimmed vacuoles (*arrows*) are observed in HE (**b**) and immunostaining for the autophagosome marker LC3b (**c**). **d** In Gomori’s trichrome, sarcoplasmic inclusions show red labeling. **e**, **f** Enzyme histochemical COX-SDH **e** and mitochondrial NADH diaforase **f** stainings show small irregular sarcoplasmic areas of reduced activity. **g** Myofibrillar protein accumulation as shown by myotilin staining is present in several fibers in DAB immunostaining, whereas desmin **h** does not accumulate in a similar manner. **i**–**l** In p62/SQSTM1, ubiquitin and SMI-31 stainings, multiple positive protein inclusions and punctate labeling are observed in nearly all fibers, whereas TDP-43 staining remains negative. Panels **f**–**l** are serial sections. Scale bars = 100 µm

**Fig. 4:**
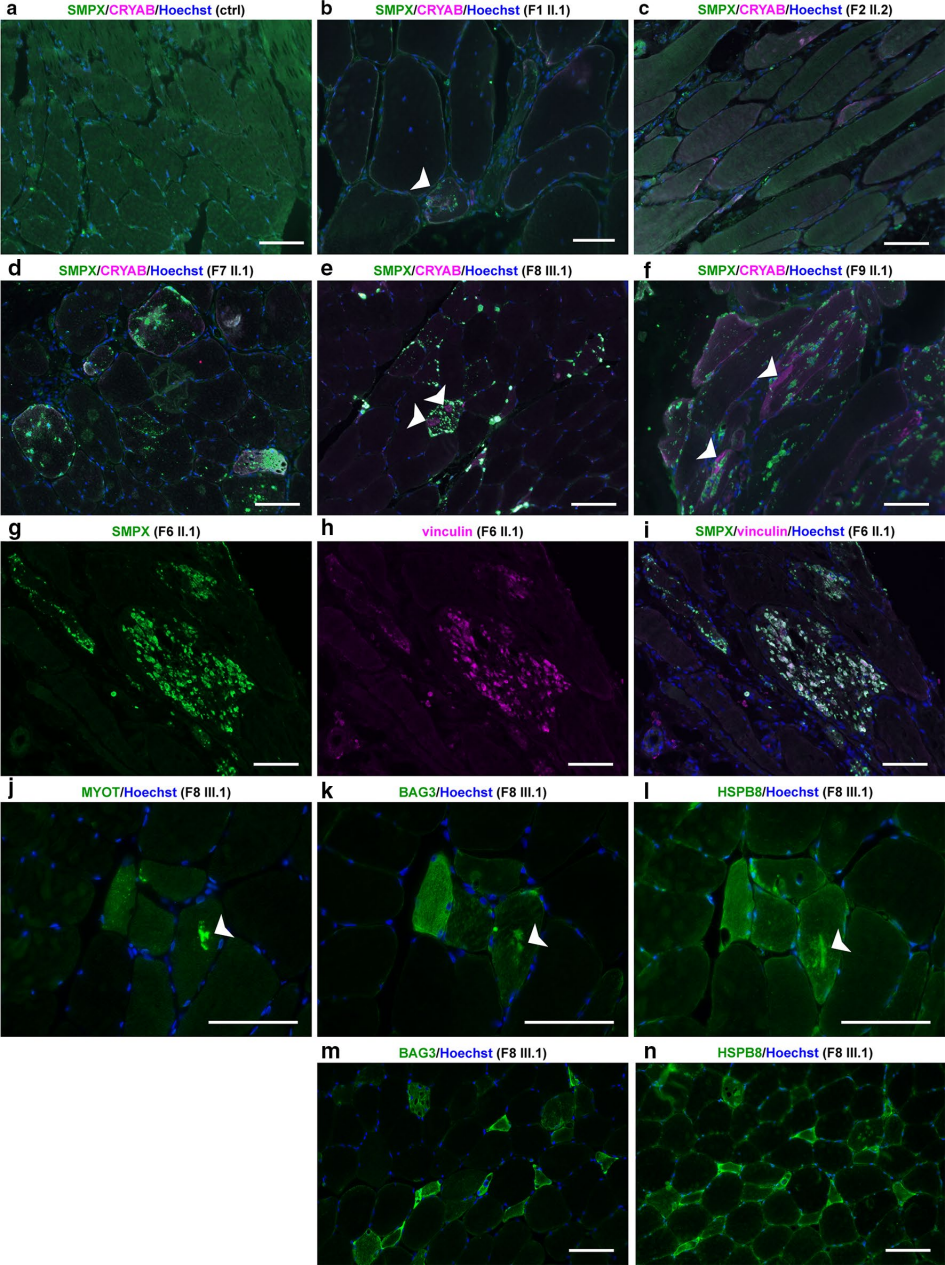
Immunofluorescence microscopy. **a**–**f** Immunofluorescent double staining; merged images are shown, SMPX is *green* and CRYAB is *magenta*. In control skeletal muscle (ctrl) free of neuromuscular disease, SMPX shows diffuse cytoplasmic and focal subsarcolemmal staining pattern. Muscle biopsy of F1 II.1 **b** shows moderate SMPX accumulation in a single fiber (*white arrowhead*), whereas in patient F2 II.2 **c** there is no SMPX accumulation. In probands from families F7, F8 and F9 (**d**–**f**), multiple SMPX-positive sarcoplasmic inclusions are present in several fibers. In F8 III.2 (**e**) and F9 II.1 (**f**), separate myofibrillar CRYAB accumulation is observed (*white arrowheads*), which does not co-localize with SMPX labeling. **g**–**i** Double immunostaining of F6 II.1. Single channel and merged images are shown; SMPX is *green*, vinculin is *magenta*. SMPX-positive protein inclusions (**g**) are also positive for vinculin (**h**), merged image (**i**). **j**–**l**; serial sections Myofibrillar accumulation (*arrow*) is positive for myotilin (**j**), and also for the CASA proteins BAG3 (**k**) and HSPB8 (**l**). **m**–**n**; serial sections) Several (atrophic) fibers show sarcoplasmic up-regulation of both BAG3 (**m**) and (**n**) HSPB8. Scale bars = 100 µm

Histopathological analysis of muscle biopsies from our patients (Table 1) showed general myopathic changes of variable severity, including fiber size variation, internal nuclei, rimmed vacuolar pathology and sarcoplasmic inclusions, which stained positive with antibodies against SMPX, SQSTM1/p62, SMI-31, ubiquitin, and the known SMPX-interacting protein vinculin (Figs. 3, 4). The protein inclusions showed sarcoplasmic and subsarcolemmal localization. Neither SMPX-positive protein inclusions, nor other SMPX accumulation was observed in any other disease control myopathies with rimmed vacuolar and/or myofibrillar pathology (2 × HMERF, 2 × sIBM, 1 × LGMD1D, 1 × WDM, and 1 × MFM with unknown etiology, data not shown). We observed more sarcoplasmic inclusions and SMPX accumulation pathology in the older patients with the N-terminal SMPX mutations (p.P7T, p.A13V and p.P27A) compared to the patients with the p.S78N mutation, located close to the SMPX C-terminus. Menadione staining in patient F9 II.1 remained negative (not shown).

**Fig. 5:**
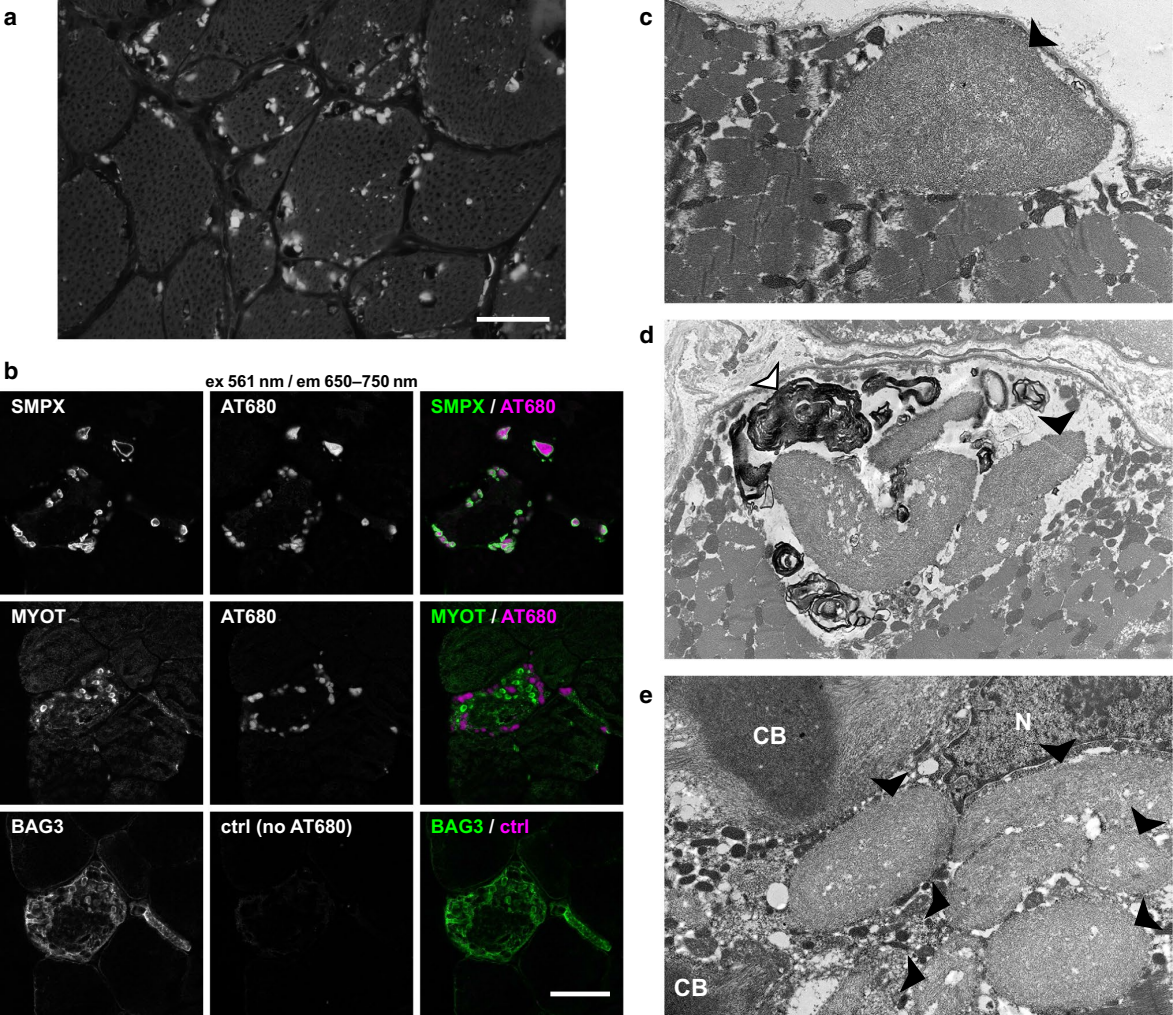
SMPX aggregation in patient muscle. **a** Congo red staining in fluorescence microscopy using Texas red filter showing abundant positive sarcoplasmic inclusions in muscle biopsy from patient F9 II.1 (scale bar = 50 µm). **b** Confocal sections of a muscle biopsy from patient F8 III.1. Immunostaining of SMPX or myotilin (*green*) combined with Amytracker 680 (AT680, *magenta*) shows Amytracker fluorescence in SMPX inclusions. A serial section of the same fiber shows no signal when Amytracker is omitted (BAG3/ctrl), showing that the protein inclusions are not autofluorescent. **c**–**e** EM findings of patient F9 II.1. **c** One large subsarcolemmal protein inclusion with accumulation of filamentous amyloid-like material (*black arrowhead*). **d** Myeloid bodies (*white arrowhead*) surrounding sarcoplasmic inclusions (*black arrowhead*). **e** Two classic cytoplasmic bodies (CB) with radiating filaments, together with six individual sarcoplasmic inclusions (*black arrowheads*) and nucleus (N)

The inclusions and protein accumulations were heterogeneous. In Herovici staining, two kinds of protein aggregates were seen: orange-red stained bodies and other protein aggregates showing dark staining (not shown). Gomori’s trichrome showed pale blue protein inclusions, dark blue granular staining, and some protein inclusions with red staining (Fig. 3d). In addition, we observed less prominent myofibrillar pathology immunoreactive for myotilin and αB-crystallin. The myofibrillar lesions showed reactivity for the CASA complex constituents BAG3 and HSPB8, both of which also accumulated in a proportion of fibers containing SMPX inclusions (Fig. 4k, l). There was no marked desmin accumulation, and TDP-43 did not show any positive immunolabeling (Fig. 3h, l). Summary of the most relevant immunohistochemical findings in sarcoplasmic inclusions vs. myofibrillar lesions is presented in Supplementary Table 1 (online resource). The sarcoplasmic inclusions in patient F9 II.1 muscle showed Congo red fluorescence when viewed with fluorescent microscope using Texas red filter, suggesting amyloid-like aggregation (Fig. 5a). In polarized light microscopy, the protein inclusions showed apple-green birefringence (not shown). Similarly, sarcoplasmic inclusions in patient F8 III.1 stained positive with the amyloid dye Amytracker 680 (Fig. 5b).

EM of patient muscles did not reveal major disorganization of sarcomeric structures; M-bands were intact whereas the alignment of Z-discs was irregular (not shown). In EM analysis of F9 II.1, some sarcoplasmic inclusions were distinct with sizes in the range of 1–5 µm, consisting of nonbranching filamentous aggregates/inclusions, and partly surrounded by autophagic myeloid structures (Fig. 5c–e). Some inclusions were compatible with cytoplasmic bodies, and sarcolemmal structures appeared intact.

### SMPX solubility analyses

**Fig. 6:**
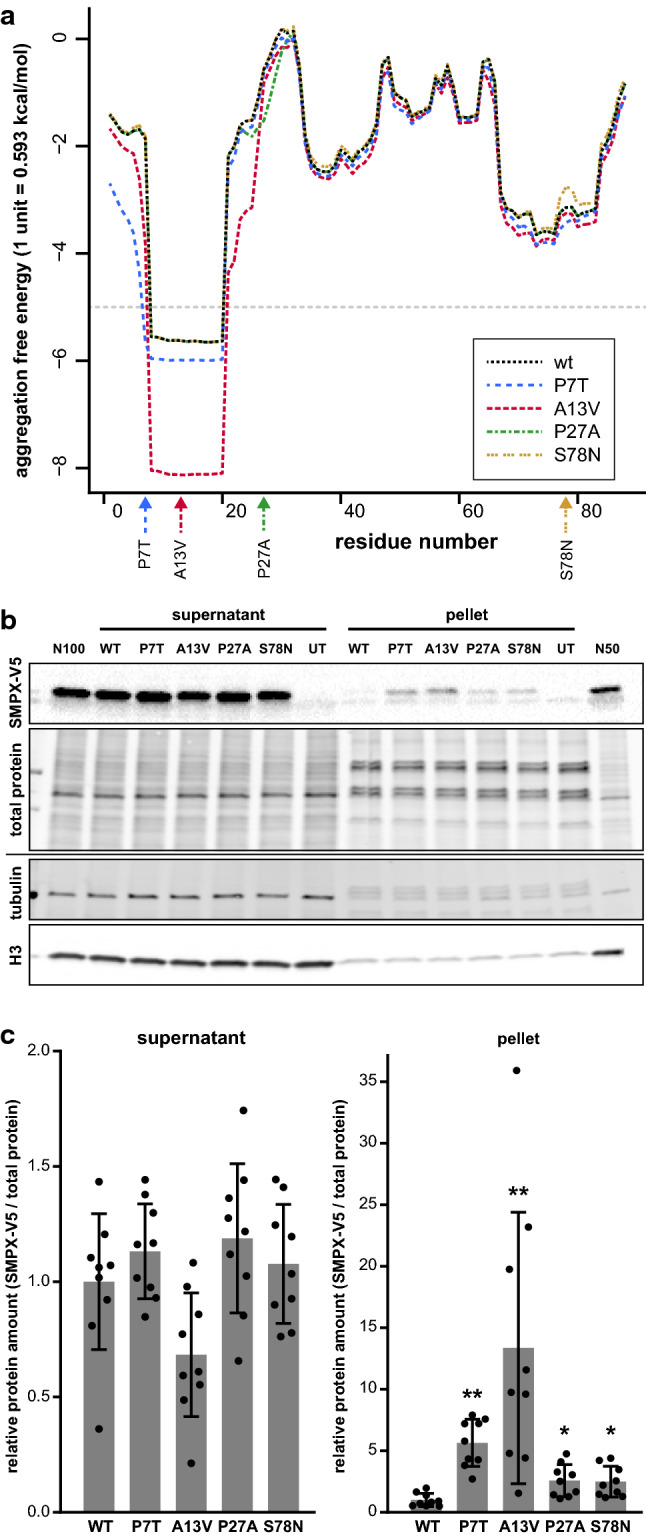
Aggregation propensity of mutant SMPX **a** In Pasta2.0 prediction, an N-terminal region of wild-type SMPX showed aggregation propensity, which was further enhanced by the p.P7T and p.A13V mutations, as indicated by the decrease in free energy. The *gray dashed line* shows the aggregation threshold of 5 PASTA units. **b** Representative fractionation experiment demonstrating reduced solubility of mutant SMPX-V5. Total protein staining of the V5 blot, and tubulin and histone 3 (H3) stainings of a separate blot are shown as loading and fractionation controls. UT, untransfected; N100 and N50, normalization samples. (c) Quantification of SMPX-V5 in supernatant and pellet fractions. The graph shows mean ± SD from three triplicate experiments (*n* = 9), normalized to total protein loading and to the mean of wild-type SMPX within each experiment. Asterisks indicate significant differences compared to WT (two-tailed Mann–Whitney U test with Bonferroni correction; * *p* < 0.05; ** *p* < 0.01)

In silico sequence analysis with Pasta2.0 [39] identified a region with high probability for parallel amyloid aggregation in the N-terminal part of wild-type SMPX, and this was further increased by the two N-terminal mutations (p.P7T and p.A13V) (Fig. 6a). Fractionation experiments of transfected HeLa cells were in excellent agreement with the prediction; they supported clearly reduced solubility of SMPX p.P7T and p.A13V, whereas the C-terminal mutations showed a minor yet significant effect (Fig. 6b, c).

### Cell culture studies and SG dynamics

To study if mutations affect the subcellular localization of SMPX, we performed IF microscopy on cultured cells. Both SMPX-V5 expressed in HeLa cells and Myc-SMPX expressed in C2C12 myoblasts showed cytoplasmic and nuclear localization, with patterns ranging from mostly diffuse to filamentous/granular/fibrillar, but no obvious differences between wild-type and mutant constructs (Supplementary Fig. 1, online resource). Some cells showed SMPX enrichment at cell edges or focal adhesions consistent with previous studies [29, 35]. We also aimed at studying SMPX localization in C2C12 myotubes, but both transient and stable transfection strategies produced few well-developed myotubes with sufficient SMPX expression levels. The small number of analyzed myotubes did not reveal localization differences between wild-type and mutant constructs (Supplementary Fig.1, online resource).

**Fig. 7:**
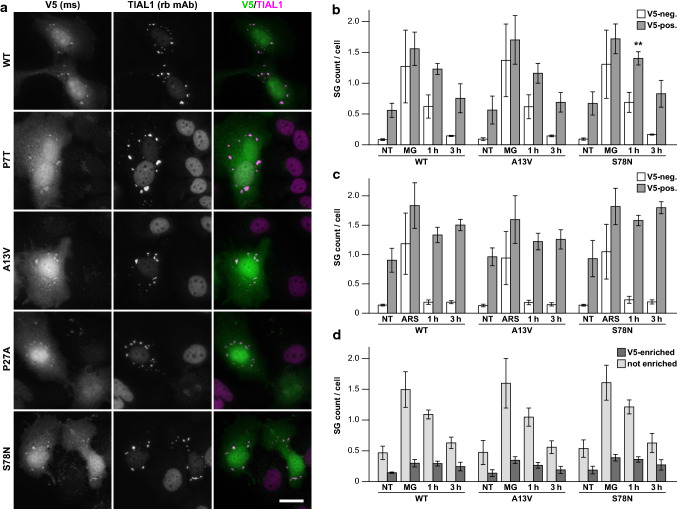
Stress granules. **a** HeLa cells showed cytoplasmic SMPX-V5 foci that were positive for TIAL1. Scale bar = 20 µm. For other SG components in non-treated and MG132-treated cells, see Supplementary Fig. 2 (online resource). **b**, **c** Quantification of TIAL1 SGs in cells positive for SMPX-V5 (*gray bars*) and V5-negative cells in the same wells (*white bars*) after MG132 (**b**) or arsenite (**c**) stress. NT, non-treated; MG, 20 µM MG132 2 h; ARS, 500 µM sodium arsenite 45 min; R1/R3, recovery of 1 h / 3 h after stress. The graphs show mean ± SD of group means from 4 experiments. Asterisks indicates a significant difference to WT according to Dunnett’s multiple comparisons test (*p* = 0.0038; mean diff. –0.1737; 95% CI –0.2424 to –0.1050). For details, see Supplementary Tables 2–3 (online resource). **d** Delayed recovery of SMPX-enriched SGs. SGs from the V5-positive cells in (**b**) were classified as V5-enriched (*dark bars*) or non-enriched (*light bars*) and expressed as SG number per cell. For details, see Supplementary Fig. 2 and Supplementary Table 4 (online resource)

Remarkably, some transfected HeLa cells showed localization of SMPX-V5 to stress granules (SG), ribonucleoprotein foci that form upon cellular stress by liquid–liquid phase separation [2]. These stained variably positive for several SG components (TIA1, TIAL1, G3BP, eIF3 and HNRNPA1) as well as the amyloid oligomer antibody A11, and suppressed protein synthesis further suggested that they are bona fide SGs (Fig. 7a, Supplementary Fig. 2). However, even under stress conditions, most of SMPX-V5 was not localized in SGs, and not all SGs in transfected cells contained visible SMPX-V5. Similarly, in C2C12 myoblasts subjected to heat shock, Myc-SMPX showed infrequent localization to SGs (Supplementary Fig. 2, online resource).

Diseases such as multisystem proteinopathy (MSP), amyotrophic lateral sclerosis, frontotemporal dementia and myopathies are associated with impaired SG dynamics or clearance [11, 16, 19, 20, 23]. To study how SMPX affects SG dynamics in transfected HeLa cells, we performed automated image analysis of TIAL1-positive SGs in non-stressed cells, immediately after stress treatments (proteasome inhibition with MG132 or oxidative stress with sodium arsenite), and after recovery. To these studies, we included p.A13V and p.S78N as examples of mutations with higher and lower effects on SMPX solubility. Compared to SMPX-negative cells, SMPX-expressing cells showed a higher proportion of SG-containing cells and increased number of SGs per cell, both in baseline and during recovery, indicating that SMPX can affect SG dynamics (Fig. 7b–c, Supplementary Fig. 2, Supplementary Tables 2, 3). Wild-type and mutant SMPX constructs did not show a dramatic difference in this experimental setup, although a significant difference in SG number per cell was seen between wild-type and p.S78N in 1-h recovery after MG132 stress, and a similar trend was apparent in arsenite experiments. Moreover, SGs enriched for SMPX-V5 showed delayed recovery after MG132 stress (Fig. 6d, Supplementary Table 4).

## Discussion

Our results demonstrate that missense mutations in *SMPX* cause a previously unidentified muscle disease, a distal myopathy with some amyloidogenic characteristics of the mutant protein. Patients have a characteristic clinical phenotype of adult-onset progressive loss of muscle tissue, first in the forearm and lower legs with later involvement of other skeletal muscles but sparing the heart muscle.

SMPX is also expressed in the hair cells of the inner ear [13, 34, 36], and variants causing a loss-of-function cause hereditary progressive non-syndromic hearing loss (NSHL, OMIM 300066) [1, 3, 7, 13, 22, 27, 28, 36] without reported skeletal myopathy. In our families, in contrast, affected adult males examined in their 6th–8th decades showed no hearing loss and neither reported any hearing impairment in their relatives. Interestingly, the first *SMPX* missense mutation causing hearing loss was recently described [10]. Replacing the C-terminal glutamine residue of SMPX with glutamate, this mutation creates a C-degron motif EE that is predicted to destabilize the protein [17], likely resulting in a hypomorphic allele pathomechanistically similar to the other hearing-loss mutations.

Our findings suggest that the missense variants described here cause muscle disease through a gain-of-function mechanism of mutant SMPX, contrasting with the loss-of-function mutations associated with hearing loss. The pathomechanism appears to be based mainly on aggregation of the mutant protein. The predicted and experimentally confirmed effect of the mutations on protein solubility correlates with the accumulation of SMPX in sarcoplasmic inclusions in patient muscle fibers, which is specific for this disease. In addition, resemblance to other myofibrillar myopathies was observed as myotilin-positive myofibrillar lesions also contain BAG3 and HSPB8. One possibility is that the myofibrillar pathology could reflect prolonged overload of the chaperonal proteostasis machinery, including the Z-disc associated chaperone-assisted selective autophagy (CASA) complex, due to SMPX aggregation.

The observed association of SMPX with SGs may also be of interest for the pathomechanism. Normal SGs are highly dynamic and dissolve quickly when the stress is over. However, prolonged stress, mutations in SG components, and accumulation of defective ribosomal products into SGs can interfere with dynamic behavior of SGs, rendering them into insoluble aggregates that need to be actively disassembled by chaperones or removed by autophagy [6]. In this context it is of particular interest that the clinical picture of our patients resembles that caused by TIA1 mutations [11, 20]. Welander distal myopathy results from a missense change in the prion-like domain of TIA1 [11], whereas a variant affecting a nearby amino acid residue leads to an identical distal myopathy phenotype in combination with SQSTM1 mutations [20]. Both of these mutations alter the prion-like behavior of TIA1, increasing its propensity to form SGs [11, 20]. Our results suggest that SMPX has the potential to interfere with the normal dissolution of SGs, and accordingly, the difficulty to express high levels of SMPX in both HeLa and C2C12 cell culture systems may be due to translational repression. The variable abnormal changes of SG proteins in patient muscle are also compatible with possible SG involvement (Supplementary Fig. 2, online resource). However, as our experiments did not reveal major differential effects on SG dynamics between wild-type and mutant SMPX, the potential role of SGs in the pathomechanism remains unclear and should be addressed in further studies in experimental systems allowing longer-term SMPX expression.

The identification of one common founder haplotype in the Italian/Maltese families and another in the French families suggest that missense variants in *SMPX* may be a more frequent cause of distal myopathy in these European populations. In gnomAD database, four anonymous male individuals from different Northern European populations are shown to carry the very late onset mutated allele c.19C>A (p.P7T), suggesting yet another possible founder mutation. This is a further indication of this new disease being more prevalent but apparently unidentified, in part because of the frequently ‘sporadic’ nature of the late onset X-linked disorders. Several different variants identified in different countries also suggest existence of yet unknown mutations and therefore screening of the small *SMPX* gene is needed in males with unsolved MFM-like pathology.

## Supplementary Information

Below is the link to the electronic supplementary material.

Supplementary file1 (PDF 8813 KB)

## Data Availability

With the exception of sensitive patient data, all imaging and biological materials are available from the authors (or commercial providers) on request.
