# Supplementary material for: Missense mutations in small muscle protein X-linked (SMPX) cause distal myopathy with protein inclusions
Source: Acta Neuropathol. 2021 May 11;142(2):375–93. doi: 10.1007/s00401-021-02319-x (PMC8270885; doi:10.1007/s00401-021-02319-x)
Supplement: Supplementary file 1 — Supplementary file1 (PDF 8813 KB) [file 401_2021_2319_MOESM1_ESM.pdf]

Johari et al. Missense mutations in small muscle protein X-linked (SMPX) cause distal myopathy with protein inclusions

Supplementary Figure 1: Localization of SMPX constructs in cultured cells

(a) SMPX-V5 in HeLa cells (single confocal sections)
(b) Myc-SMPX in C2C12 myoblasts (single confocal sections)
(c) SMPX-V5 in C2C12 myotubes (maximum intensity projections)
(d) Myc-SMPX in C2C12 myotubes (maximum intensity projections)
The image intensity levels have been individually adjusted and are not comparable between images. Scale bars = 20 μm

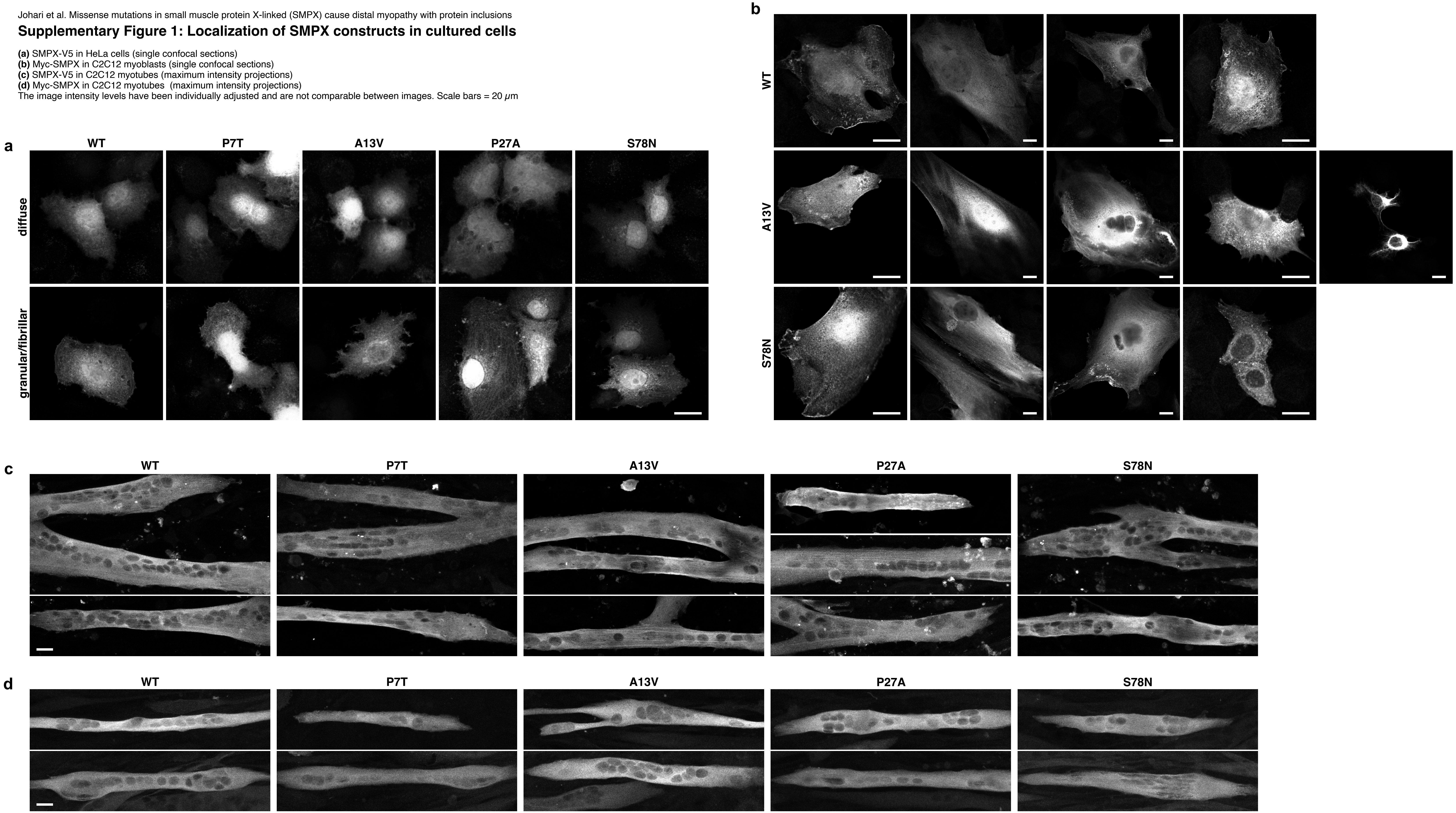

Supplementary Figure 2: SMPX and stress granules

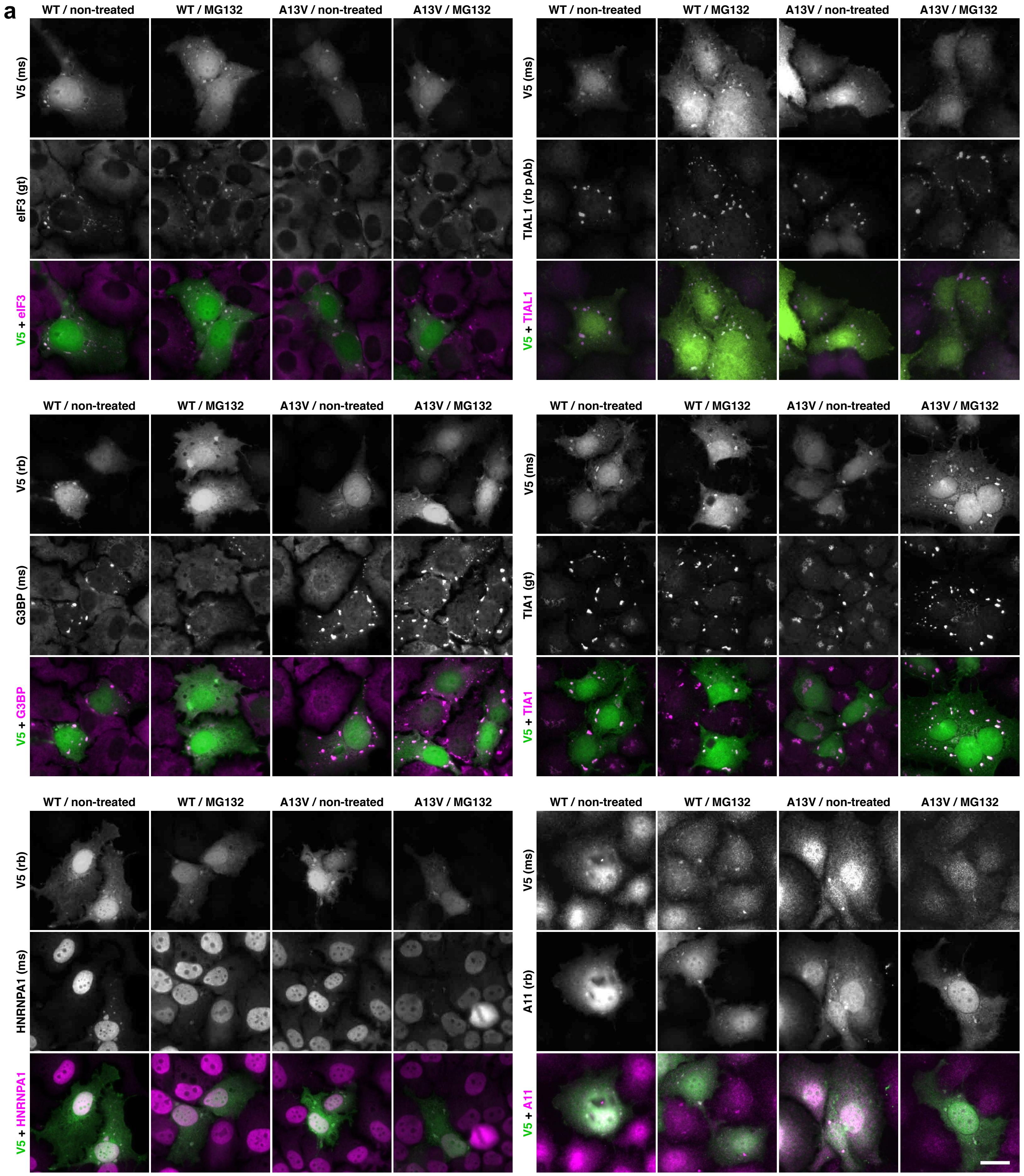

(a) Localization of SMPX-V5 to stress granules in HeLa cells. Wild-type (WT) and p.A13V SMPX-V5 were expressed in HeLa cells for 3 d. Cells were left non-treated or treated with 20 μM MG132 for 2 h prior to fixing. SMPX-containing SGs stained positive for eIF3, G3BP, TIA1, TIAL1, HNRNPA1, and the amyloid oligomer antibody A11. Scale bar = 20 μm.

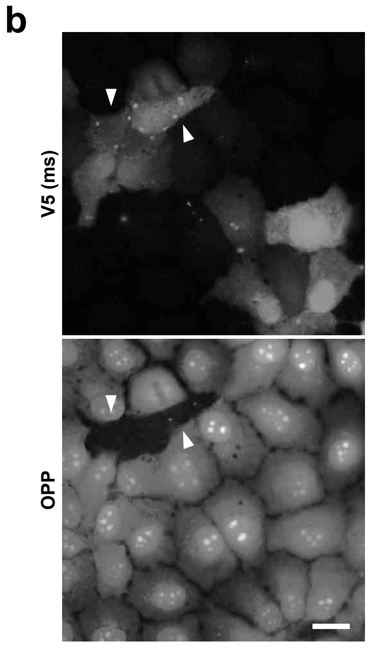

(b) Lack of OP-puromycin (OPP) incorporation indicating blocked protein synthesis in HeLa cells containing SMPX-V5 foci (arrowheads). Scale bar = 20 μm.

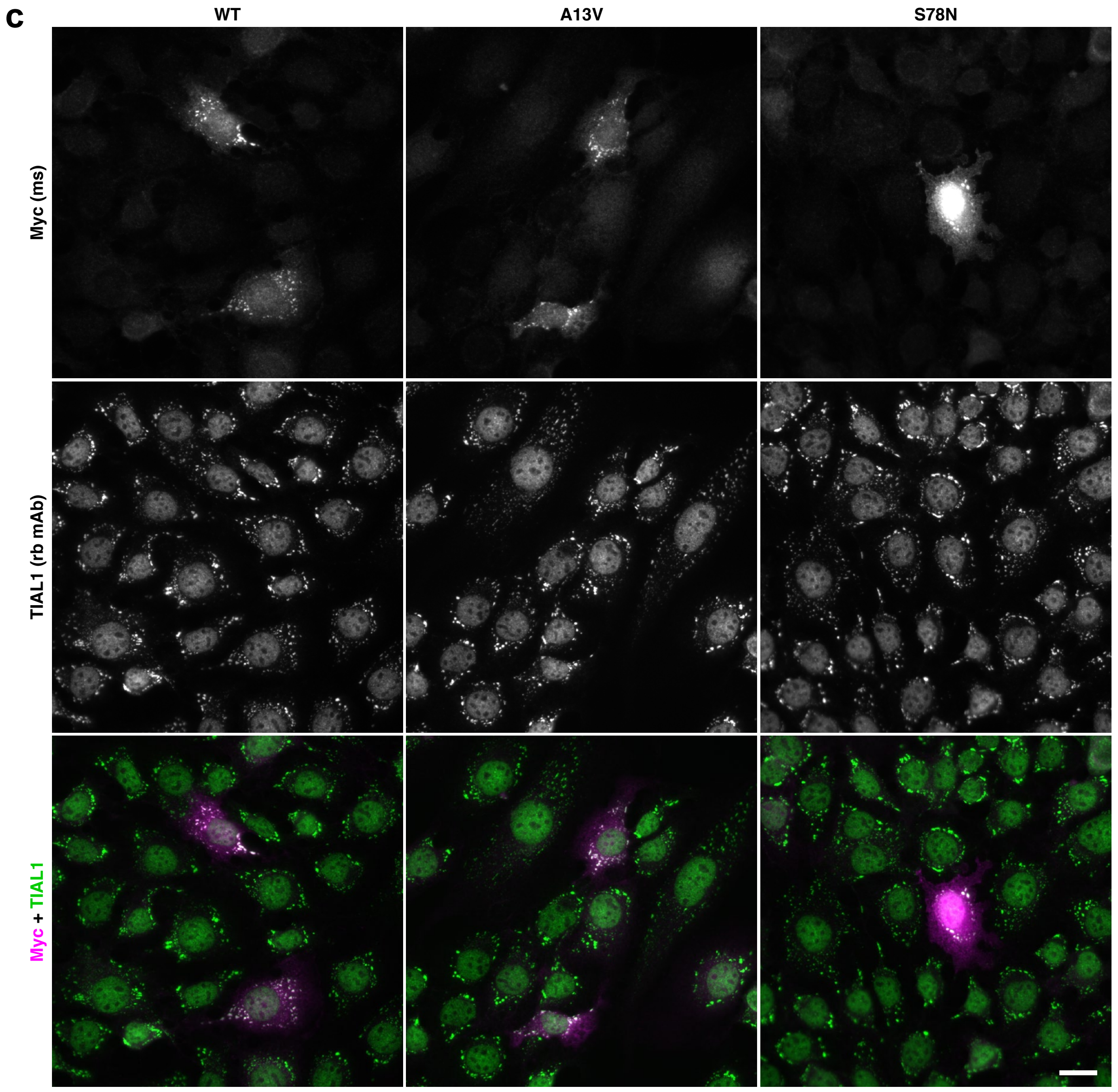

(c) Localization of Myc-SMPX cells to stress granules in C2C12 myoblasts. Wild-type or mutant Myc-SMPX was expressed in stably transfected C2C12 myoblasts for 2 d, and SG formation was induced by heat shock (1 h in 45°C) before fixing. Scale bar = 20 μm.

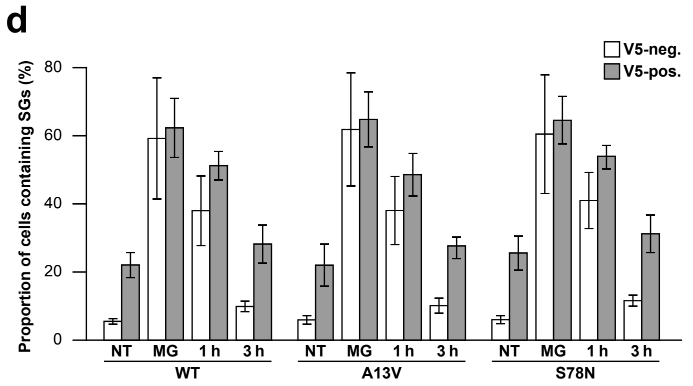

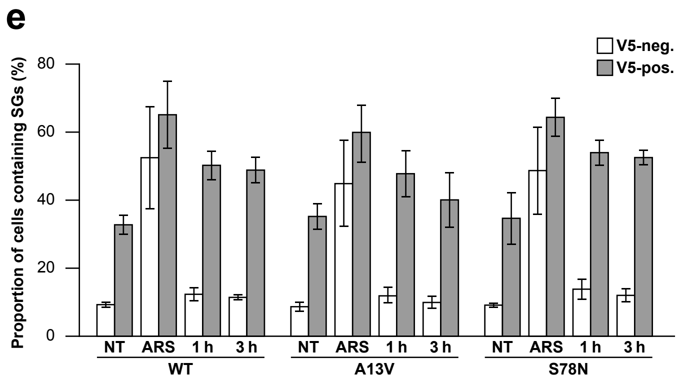

(d–e) The proportion of V5-negative and V5-positive cells containing ≥ 1 SGs in in MG132 (d) and arsenite stress (e) experiments presented in Fig. 6b–c and Supplementary tables 2–3. The graphs show mean ± SD of group means from 4 experiments.

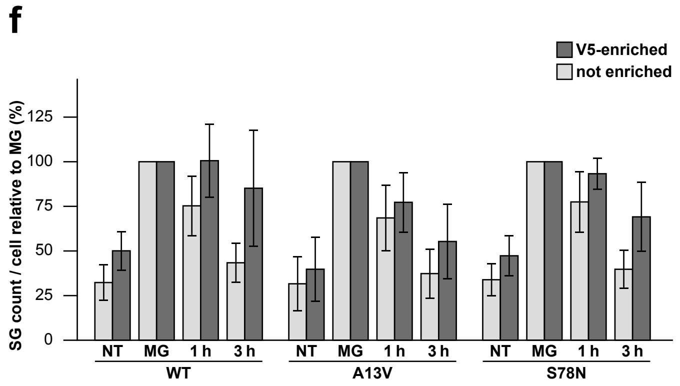

(f) Recovery of SMPX-V5-enriched and non-enriched SGs after MG132 stress. Data from Fig. 6d presented normalized to MG within each experiment. The graphs show mean ± SD of group means from 4 experiments.

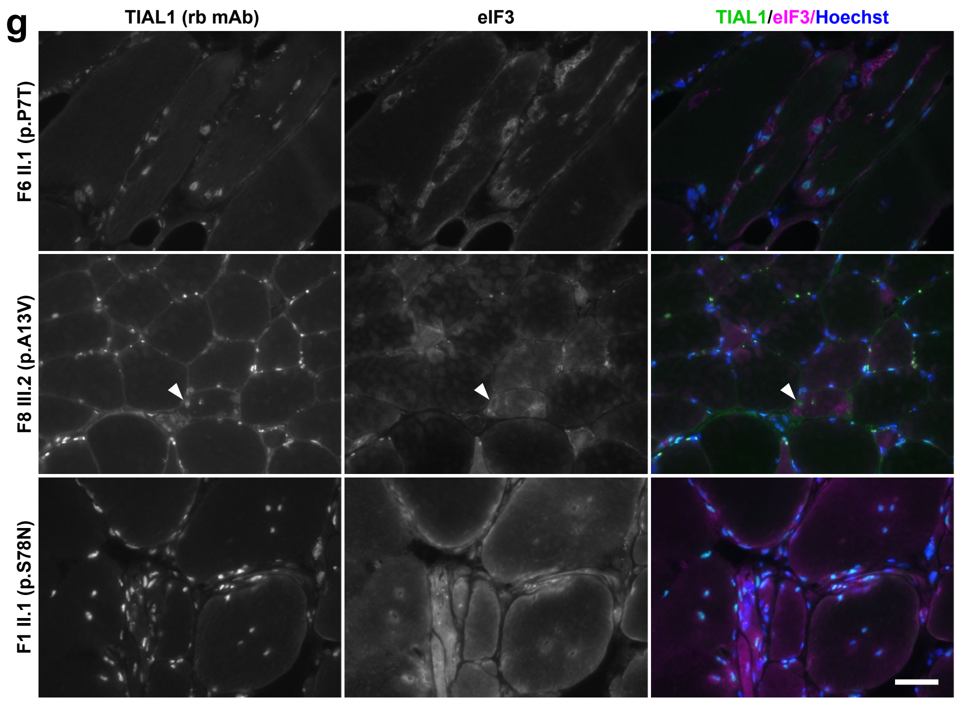

(g) IF analysis of SG components in patient muscle biopsies. In F6 II.1 and F8 III.2, accumulation of eIF3 was observed subsarcolemmally and surrounding the sarcoplasmic inclusions, a subset of which stained also positive for TIAL1 in F8 III.2 (arrowhead). In F1 II.1, an increased level of eIF3, but not TIAL1, was seen in the sarcoplasm of atrophic fibers. Scale bar = 50 μm.

Supplementary Table 1: Immunohistochemical findings in SMPX patients

Antibody	protein inclusions	MFM
SMPX	+	-
p62	+	-
Ubiquitin	+	-
TDP-43	-	-
SMI-31	+	-
Myotilin	-	+
αB-crystallin	-	+

Supplementary Table 2: SG dynamics in MG132-stressed cells

SG dynamics (Fig. 7, Suppl. Fig. 2)				exp 1	exp 2	exp 3	exp 4	sum	mean	SD
WT	NT	V5-neg. cells	cell count	5460	7189	7739	15415	35803		
			SGs/cell	0,060	0,077	0,094	0,105		0,084	0,020
			cells with SGs	4,3%	5,4%	5,8%	6,6%		5,5%	1,0%
		V5-pos. cells	cell count	866	922	986	1996	4770		
			SGs/cell	0,387	0,656	0,624	0,573		0,560	0,120
			cells with SGs	16,3%	24,1%	24,2%	23,6%		22,1%	3,9%
	MG	V5-neg. cells	cell count	6723	6336	13117	13023	39199		
			SGs/cell	1,683	1,884	0,752	0,768		1,272	0,596
			cells with SGs	74,0%	75,6%	43,5%	43,9%		59,2%	18,0%
		V5-pos. cells	cell count	1119	778	1624	1786	5307		
			SGs/cell	1,666	1,901	1,325	1,348		1,560	0,276
			cells with SGs	69,3%	70,6%	54,3%	55,1%		62,3%	8,8%
	R1	V5-neg. cells	cell count	5721	8996	12110	13673	40500		
			SGs/cell	0,870	0,595	0,396	0,625		0,622	0,194
			cells with SGs	50,5%	37,6%	25,1%	38,8%		38,0%	10,4%
		V5-pos. cells	cell count	1167	1408	1597	1987	6159		
			SGs/cell	1,136	1,281	1,165	1,339		1,230	0,095
			cells with SGs	53,9%	52,3%	44,6%	54,0%		51,2%	4,4%
	R3	V5-neg. cells	cell count	5001	10223	11856	11719	38799		
			SGs/cell	0,151	0,163	0,129	0,135		0,145	0,015
			cells with SGs	11,5%	11,1%	7,8%	9,2%		9,9%	1,7%
		V5-pos. cells	cell count	845	1439	1681	1613	5578		
			SGs/cell	0,400	0,915	0,814	0,893		0,755	0,241
			cells with SGs	20,1%	32,3%	28,1%	32,3%		28,2%	5,7%
A13V	NT	V5-neg. cells	cell count	5659	7075	10721	14137	37592		
			SGs/cell	0,056	0,081	0,105	0,124		0,092	0,030
			cells with SGs	4,0%	5,6%	6,9%	7,3%		5,9%	1,5%
		V5-pos. cells	cell count	1241	928	1555	1937	5661		
			SGs/cell	0,223	0,746	0,660	0,628		0,564	0,233
			cells with SGs	12,7%	26,7%	23,7%	25,0%		22,0%	6,4%
	MG	V5-neg. cells	cell count	6589	6522	14156	12578	39845		
			SGs/cell	1,793	1,973	0,836	0,886		1,372	0,595
			cells with SGs	75,8%	76,8%	45,4%	49,3%		61,8%	16,8%
		V5-pos. cells	cell count	1125	874	1794	1791	5584		
			SGs/cell	1,826	2,211	1,318	1,459		1,703	0,400
			cells with SGs	70,7%	73,1%	56,4%	59,0%		64,8%	8,3%
	R1	V5-neg. cells	cell count	5941	8592	13004	12526	40063		
			SGs/cell	0,797	0,570	0,357	0,748		0,618	0,200
			cells with SGs	47,2%	37,0%	24,2%	44,0%		38,1%	10,2%
		V5-pos. cells	cell count	1090	1317	1728	1825	5960		
			SGs/cell	1,154	1,160	0,962	1,369		1,161	0,166
			cells with SGs	52,5%	48,0%	39,6%	54,2%		48,6%	6,5%
	R3	V5-neg. cells	cell count	5320	9636	11614	12047	38617		
			SGs/cell	0,155	0,167	0,128	0,127		0,144	0,020
			cells with SGs	12,3%	12,2%	7,6%	8,5%		10,2%	2,4%
		V5-pos. cells	cell count	1096	1421	1499	1815	5831		
			SGs/cell	0,450	0,755	0,817	0,739		0,690	0,164
			cells with SGs	22,9%	30,5%	28,8%	28,4%		27,7%	3,3%
S78N	NT	V5-neg. cells	cell count	4985	4109	4183	13305	26582		
			SGs/cell	0,058	0,099	0,105	0,110		0,093	0,024
			cells with SGs	4,1%	5,9%	7,1%	7,0%		6,0%	1,4%
		V5-pos. cells	cell count	904	598	564	1832	3898		
			SGs/cell	0,423	0,895	0,707	0,663		0,672	0,194
			cells with SGs	19,2%	31,8%	26,6%	24,7%		25,6%	5,2%
	MG	V5-neg. cells	cell count	7455	8130	8912	8749	33246		
			SGs/cell	1,654	1,900	0,964	0,716		1,309	0,560
			cells with SGs	73,3%	77,5%	50,0%	41,3%		60,5%	17,6%
		V5-pos. cells	cell count	1343	1135	1110	1228	4816		
			SGs/cell	1,695	2,075	1,594	1,518		1,720	0,247
			cells with SGs	68,1%	73,0%	58,9%	58,2%		64,6%	7,2%
	R1	V5-neg. cells	cell count	7584	10373	12067	12624	42648		
			SGs/cell	0,892	0,633	0,495	0,734		0,689	0,167
			cells with SGs	50,5%	39,7%	30,3%	43,4%		41,0%	8,4%
		V5-pos. cells	cell count	1241	1518	1684	1892	6335		
			SGs/cell	1,315	1,418	1,324	1,559		1,404	0,113
			cells with SGs	56,3%	54,2%	48,8%	56,7%		54,0%	3,6%
	R3	V5-neg. cells	cell count	6994	7254	7896	12051	34195		
			SGs/cell	0,168	0,179	0,171	0,146		0,166	0,014
			cells with SGs	13,0%	13,1%	10,8%	9,5%		11,6%	1,8%
		V5-pos. cells	cell count	1176	1072	1146	1772	5166		
			SGs/cell	0,512	1,027	0,846	0,931		0,829	0,224
			cells with SGs	24,1%	37,9%	30,1%	32,7%		31,2%	5,7%

Supplementary Table 3: SG dynamics in arsenite-stressed cells

SG dynamics (Fig. 7, Suppl. Fig. 2)				exp 1	exp 2	exp 3	exp 4	sum	mean	SD
WT	NT	V5-neg. cells	cell count	5762	9723	4704	5913	26102		
			SGs/cell	0,111	0,132	0,149	0,160		0,138	0,021
			cells with SGs	8,0%	9,4%	9,5%	10,3%		9,3%	0,9%
		V5-pos. cells	cell count	707	757	538	561	2563		
			SGs/cell	0,622	0,871	1,097	1,021		0,903	0,209
			cells with SGs	28,4%	32,9%	34,9%	34,8%		32,8%	3,0%
	ARS	V5-neg. cells	cell count	14903	17468	12860	10240	55471		
			SGs/cell	1,434	1,802	0,662	0,838		1,184	0,528
			cells with SGs	61,7%	68,7%	36,6%	42,9%		52,5%	15,1%
		V5-pos. cells	cell count	1841	1593	1485	739	5658		
			SGs/cell	2,083	2,204	1,719	1,330		1,834	0,394
			cells with SGs	71,8%	74,9%	60,6%	53,2%		65,1%	10,0%
	R1	V5-neg. cells	cell count	8753	14609	10501	13893	47756		
			SGs/cell	0,244	0,157	0,150	0,201		0,188	0,044
			cells with SGs	15,2%	10,9%	10,5%	12,8%		12,3%	2,2%
		V5-pos. cells	cell count	656	1047	1030	1128	3861		
			SGs/cell	1,299	1,363	1,171	1,499		1,333	0,137
			cells with SGs	55,2%	46,6%	46,5%	52,7%		50,2%	4,4%
	R3	V5-neg. cells	cell count	5404	13076	na	8034	26514		
			SGs/cell	0,220	0,157	na	0,188		0,188	0,032
			cells with SGs	12,3%	10,5%	na	11,5%		11,4%	0,9%
		V5-pos. cells	cell count	498	1040	na	601	2139		
			SGs/cell	1,506	1,400	na	1,602		1,503	0,101
			cells with SGs	52,6%	44,8%	na	49,1%		48,8%	3,9%
A13V	NT	V5-neg. cells	cell count	9730	9674	9499	8364	37267		
			SGs/cell	0,097	0,136	0,167	0,125		0,131	0,029
			cells with SGs	7,2%	9,1%	10,6%	7,9%		8,7%	1,5%
		V5-pos. cells	cell count	811	711	879	579	2980		
			SGs/cell	0,732	1,020	1,003	1,090		0,961	0,157
			cells with SGs	29,5%	38,4%	35,8%	37,1%		35,2%	4,0%
	ARS	V5-neg. cells	cell count	15268	13731	21500	11192	61691		
			SGs/cell	0,808	1,615	0,592	0,746		0,940	0,459
			cells with SGs	42,4%	63,4%	34,3%	39,3%		44,9%	12,8%
		V5-pos. cells	cell count	1216	1042	1562	549	4369		
			SGs/cell	1,433	2,194	1,249	1,508		1,596	0,413
			cells with SGs	57,4%	72,5%	53,0%	56,8%		59,9%	8,6%
	R1	V5-neg. cells	cell count	9689	10044	13097	15698	48528		
			SGs/cell	0,243	0,137	0,170	0,180		0,182	0,044
			cells with SGs	15,2%	9,2%	11,4%	11,7%		11,9%	2,5%
		V5-pos. cells	cell count	599	607	974	836	3016		
			SGs/cell	1,419	1,082	1,142	1,240		1,221	0,147
			cells with SGs	58,1%	42,7%	45,5%	44,9%		47,8%	7,0%
	R3	V5-neg. cells	cell count	10624	8169	na	6669	25462		
			SGs/cell	0,176	0,108	na	0,150		0,145	0,034
			cells with SGs	11,7%	7,9%	na	10,2%		9,9%	2,0%
		V5-pos. cells	cell count	801	508	na	401	1710		
			SGs/cell	1,449	1,122	na	1,202		1,258	0,171
			cells with SGs	49,4%	36,6%	na	34,2%		40,1%	8,2%
S78N	NT	V5-neg. cells	cell count	8830	6207	6935	7198	29170		
			SGs/cell	0,109	0,151	0,153	0,133		0,136	0,020
			cells with SGs	8,4%	10,0%	9,6%	8,5%		9,1%	0,8%
		V5-pos. cells	cell count	1214	790	832	785	3621		
			SGs/cell	0,528	0,834	1,210	1,149		0,930	0,315
			cells with SGs	24,7%	32,5%	42,2%	39,2%		34,7%	7,8%
	ARS	V5-neg. cells	cell count	11305	15189	22526	12082	61102		
			SGs/cell	0,964	1,731	0,662	0,834		1,048	0,472
			cells with SGs	48,9%	66,6%	36,6%	42,7%		48,7%	13,0%
		V5-pos. cells	cell count	1425	1810	1556	941	5732		
			SGs/cell	1,587	2,277	1,638	1,775		1,819	0,316
			cells with SGs	62,1%	72,9%	60,9%	61,4%		64,3%	5,7%
	R1	V5-neg. cells	cell count	14283	14956	12123	15193	56555		
			SGs/cell	0,322	0,187	0,219	0,184		0,228	0,065
			cells with SGs	18,2%	11,5%	13,9%	11,8%		13,8%	3,1%
		V5-pos. cells	cell count	1190	1673	1356	1284	5503		
			SGs/cell	1,679	1,551	1,626	1,463		1,580	0,094
			cells with SGs	59,1%	50,4%	54,8%	51,6%		54,0%	3,9%
	R3	V5-neg. cells	cell count	15787	11557	na	10653	37997		
			SGs/cell	0,236	0,189	na	0,152		0,192	0,042
			cells with SGs	14,3%	11,5%	na	10,2%		12,0%	2,1%
		V5-pos. cells	cell count	1692	1232	na	916	3840		
			SGs/cell	1,680	1,824	na	1,892		1,798	0,108
			cells with SGs	54,8%	50,2%	na	52,6%		52,5%	2,3%

na, omitted due to failed staining

Supplementary Table 4: Effect of SMPX-V5 enrichment on SG recovery

V5 enrichment (Fig. 7, Suppl. Fig. 2)				exp 1	exp 2	exp 3	exp 4	mean	SD
WT	NT	not V5-enr. SGs	per cell	0,308	0,556	0,542	0,462	0,467	0,114
			norm. to MG	18,8 %	30,2 %	43,0 %	37,3 %	32,3 %	10,4 %
		V5-enr. SGs	per cell	0,122	0,155	0,143	0,158	0,145	0,016
			norm. to MG	41,1 %	40,5 %	63,3 %	55,4 %	50,1 %	11,2 %
	MG	not V5-enr. SGs	per cell	1,641	1,841	1,259	1,241	1,495	0,295
			norm. to MG	100,0 %	100,0 %	100,0 %	100,0 %	100,0 %	
		V5-enr. SGs	per cell	0,298	0,383	0,226	0,286	0,298	0,065
			norm. to MG	100,0 %	100,0 %	100,0 %	100,0 %	100,0 %	
	R1	not V5-enr. SGs	per cell	1,072	1,077	1,015	1,200	1,091	0,078
			norm. to MG	65,3 %	58,5 %	80,6 %	96,7 %	75,3 %	17,0 %
		V5-enr. SGs	per cell	0,237	0,347	0,291	0,296	0,293	0,045
			norm. to MG	79,5 %	90,5 %	128,6 %	103,8 %	100,6 %	21,1 %
	R3	not V5-enr. SGs	per cell	0,491	0,716	0,624	0,684	0,629	0,099
			norm. to MG	29,9 %	38,9 %	49,6 %	55,1 %	43,4 %	11,2 %
		V5-enr. SGs	per cell	0,133	0,283	0,268	0,296	0,245	0,076
			norm. to MG	44,5 %	73,8 %	118,7 %	103,6 %	85,2 %	32,9 %
A13V	NT	not V5-enr. SGs	per cell	0,187	0,647	0,565	0,498	0,474	0,201
			norm. to MG	10,4 %	31,3 %	47,0 %	37,8 %	31,6 %	15,6 %
		V5-enr. SGs	per cell	0,047	0,165	0,167	0,171	0,137	0,060
			norm. to MG	14,6 %	37,5 %	54,4 %	52,5 %	39,8 %	18,4 %
	MG	not V5-enr. SGs	per cell	1,805	2,066	1,200	1,318	1,597	0,408
			norm. to MG	100,0 %	100,0 %	100,0 %	100,0 %	100,0 %	
		V5-enr. SGs	per cell	0,319	0,439	0,306	0,326	0,348	0,062
			norm. to MG	100,0 %	100,0 %	100,0 %	100,0 %	100,0 %	
	R1	not V5-enr. SGs	per cell	1,068	1,034	0,857	1,231	1,048	0,153
			norm. to MG	59,2 %	50,0 %	71,4 %	93,4 %	68,5 %	18,8 %
		V5-enr. SGs	per cell	0,224	0,281	0,223	0,333	0,265	0,053
			norm. to MG	70,1 %	63,9 %	72,8 %	102,2 %	77,3 %	17,0 %
	R3	not V5-enr. SGs	per cell	0,396	0,610	0,631	0,597	0,558	0,109
			norm. to MG	21,9 %	29,5 %	52,6 %	45,3 %	37,3 %	14,1 %
		V5-enr. SGs	per cell	0,095	0,212	0,241	0,210	0,190	0,065
			norm. to MG	29,7 %	48,2 %	78,7 %	64,7 %	55,3 %	21,1 %
S78N	NT	not V5-enr. SGs	per cell	0,346	0,689	0,601	0,507	0,536	0,147
			norm. to MG	20,5 %	34,9 %	42,1 %	38,2 %	33,9 %	9,4 %
		V5-enr. SGs	per cell	0,107	0,264	0,163	0,216	0,188	0,068
			norm. to MG	33,1 %	56,5 %	43,0 %	56,7 %	47,3 %	11,5 %
	MG	not V5-enr. SGs	per cell	1,692	1,974	1,429	1,329	1,606	0,289
			norm. to MG	100,0 %	100,0 %	100,0 %	100,0 %	100,0 %	
		V5-enr. SGs	per cell	0,325	0,468	0,379	0,380	0,388	0,059
			norm. to MG	100,0 %	100,0 %	100,0 %	100,0 %	100,0 %	
	R1	not V5-enr. SGs	per cell	1,212	1,215	1,067	1,358	1,213	0,119
			norm. to MG	71,6 %	61,5 %	74,6 %	102,2 %	77,5 %	17,4 %
		V5-enr. SGs	per cell	0,287	0,388	0,388	0,378	0,360	0,049
			norm. to MG	88,4 %	82,9 %	102,2 %	99,5 %	93,3 %	9,1 %
	R3	not V5-enr. SGs	per cell	0,415	0,802	0,618	0,673	0,627	0,161
			norm. to MG	24,5 %	40,6 %	43,2 %	50,6 %	39,8 %	11,0 %
		V5-enr. SGs	per cell	0,138	0,310	0,309	0,328	0,271	0,089
			norm. to MG	42,4 %	66,2 %	81,4 %	86,4 %	69,1 %	19,7 %
